# StREM1.3 REMORIN Protein Plays an Agonistic Role in Potyvirus Cell-to-Cell Movement in *N. benthamiana*

**DOI:** 10.3390/v14030574

**Published:** 2022-03-10

**Authors:** Marion Rocher, Vincent Simon, Marie-Dominique Jolivet, Luc Sofer, Anne-Flore Deroubaix, Véronique Germain, Sébastien Mongrand, Sylvie German-Retana

**Affiliations:** 1 Laboratoire de Biogenèse Membranaire, UMR 5200, CNRS Université Bordeaux, 71 Av. E. Bourlaux, 33140 Villenave d’Ornon, France; marion.r94@laposte.net (M.R.); marie-dominique.jolivet@u-bordeaux.fr (M.-D.J.); deroubaix.anneflore@orange.fr (A.-F.D.); veronique.germain@u-bordeaux.fr (V.G.); sebastien.mongrand@u-bordeaux.fr (S.M.); 2 UMR 1332 Biologie du Fruit et Pathologie, INRAE Université Bordeaux, 71 Av. E. Bourlaux, CS20032, CEDEX, 33882 Villenave d’Ornon, France; vincent.simon@inrae.fr (V.S.); luc.sofer@inrae.fr (L.S.)

**Keywords:** REMORIN, StREM1.3, *Nicotiana benthamiana*, potyvirus, TuMV, PVA, movement, cylindrical inclusion, CI, callose

## Abstract

REMORIN proteins belong to a plant-specific multigene family that localise in plasma membrane nanodomains and in plasmodesmata. We previously showed that in *Nicotiana benthamiana*, group 1 StREM1.3 limits the cell-to-cell spread of a potexvirus without affecting viral replication. This prompted us to check whether an effect on viral propagation could apply to potyvirus species *Turnip mosaic virus* (TuMV) and *Potato virus A* (PVA). Our results show that StREM1.3 transient or stable overexpression in transgenic lines increases potyvirus propagation, while it is slowed down in transgenic lines underexpressing endogenous NbREMs, without affecting viral replication. TuMV and PVA infection do not alter the membranous localisation of StREM1.3. Furthermore, StREM1.3-membrane anchoring is necessary for its agonist effect on potyvirus propagation. StREM1.3 phosphocode seems to lead to distinct plant responses against potexvirus and potyvirus. We also showed that StREM1.3 interacts in yeast and in planta with the key potyviral movement protein CI (cylindrical inclusion) at the level of the plasma membrane but only partially at plasmodesmata pit fields. TuMV infection also counteracts StREM1.3-induced plasmodesmata callose accumulation at plasmodesmata. Altogether, these results showed that StREM1.3 plays an agonistic role in potyvirus cell-to-cell movement in *N. benthamiana*.

## 1. Introduction

To invade the whole plant after replication, plant viruses must move intracellularly to reach the plasmodesmata (PD), the symplasmic channels between plant cells that are the gateway for this movement. Viruses cross PD to pass to neighbouring cells and finally enter into sieve elements [1]. They are then passively transported within the source-to-sink flow of photoassimilates and are unloaded from sieve elements into sink tissues [2]. Plant viruses use an active mechanism to move from the site of replication within the cell to the PD for cell-to-cell movement. The plant virus genome encodes a class of proteins called movement proteins (MPs) that interact with host proteins to modify the PD size exclusion limit for cell-to-cell transportation of the viral genome [3]. Viral MPs mediate interactions between viral nucleic acids and host proteins to hijack PD via two distinct mechanisms. One mechanism, exemplified by the 30K-type MP of *Tobacco mosaic virus* (TMV), may be common to all viruses that do not move as intact virions, including the triple gene block proteins (TGBps) of potexviruses and hordeiviruses. Those viruses are considered as “non-tubule-forming viruses”, which do not morphologically drastically alter PD. Another group of viruses, exemplified by members of the families Secoviridae, Bromoviridae, and Caulimoviridae, move as intact virions. The MPs of these viruses form large tubules that insert into PD to allow the transport of intact viruses between cells. Those viruses are considered “tubule-forming viruses”, which induce drastic alteration in PD, with the elimination of the desmotubule.

The cell-to-cell movement mechanism of potyviruses, one of the largest genera of plant RNA viruses responsible for serious diseases in vegetable and fruit crops [4], does not fall into any of the two previous categories; it is a very complex and still very poorly understood mechanism for potyvirus infection. In particular, no dedicated movement protein has been identified, but several viral proteins with other known functions have been reported to participate in potyvirus movement [5]. In particular, for the potyvirus species *Turnip mosaic virus* (TuMV), reverse-genetic and cellular studies showed the crucial role played by the capsid protein and three nonstructural proteins: the cylindrical inclusion helicase (CI), the “second 6K molecular-weight membrane anchoring protein” (6K2) and pretty interesting Potyviridae ORF (P3N-PIPO) [6,7,8,9]. The 6K2 of TuMV is a membrane protein involved in endomembrane rearrangements of the endoplasmic reticulum (ER) for the generation of membranous viral vesicles compartments, important for replication [10], as well as for intracellular and intercellular movement [11,12,13,14]. For TuMV, such vesicles are also recruited to PD by the CI and P3N-PIPO viral proteins [7,15]. TuMV represents one of the rare examples of plant viruses that utilise the host endomembrane system to produce membranous vesicles mobile between cells, reminding of animal viruses that utilise membrane-derived vesicles for exit from infected cells and entry into healthy cells [16]. Recently, the observation in the extracellular space and cell wall of TuMV-induced vesicles suggests a novel striking mechanism for viral cell-to-cell spreading [17,18]. A few plant factors involved in potyvirus cell-to-cell movement were identified using virus-target-based approaches (such as yeast two-hybrid cDNA library screening and in planta pull-down assays using viral baits), forward-genetics, and bottom-up proteomic approaches. Those factors are diverse and correspond to PM-associated proteins targeted to PD [19], cell-wall-loosening protein [20], HSP70-related chaperones [21], proteins essential for endomembrane remodelling, endoplasmic reticulum bending, inter-organelle vesicular exchanges [13,17,22,23,24,25], and proteins involved in endocytic processes [26,27,28]. Those factors identified in diverse plant–potyvirus pathosystems were shown to be proviral movement factors. Lastly, the plant plasma membrane-associated REMORIN protein (AtREM1.2) was shown to impair potyvirus movement in *A. thaliana* [29].

REMORINS are plant-specific plasma membrane (PM) nanodomain markers involved in response to biotic and abiotic stresses [30]. REMORIN proteins present a highly conserved C-terminal domain and a divergent N-terminal domain and were classified into six phylogenetic groups [31]. The first REM protein identified in potato was called *Solanum tuberosum* REMORIN group 1 homologue 3 (hereafter StREM1.3) and belongs to group 1b REMs. Group 1b REMORIN of Solanaceae plants (namely, potato StREM, tobacco NtREM, tomato SlREM, and *N. benthamiana* NbREM) are highly homologous and they colocalise in nanodomains of the PM [32,33]. Our present study is focused on this group 1 REMORINS from Solanaceae, further designated as REMs here. Group 1 REMs are characterised by an intrinsic disordered proline-rich N-terminal domain that has many predicted phosphorylation sites [31,34]. REM proteins are proposed as nanodomain-organising proteins [35], and REM oligomerisation is highly important for its stability and function [30]. REMs are strongly embedded in the inner leaflet of the PM by an unconventional mechanism involving anionic lipids and sterols, the protein-lipid-binding motif being called REMORIN C-terminal Anchor (REM–CA) [36]. The presence of group 1 REMs in PM nanodomains was associated with an increased effect on the accumulation of callose at PD pit fields, suggesting that REM proteins also regulate cell-to-cell communication through PD [32,35]. The discovery of group 1 REMs in a proteomic analysis of *A. thaliana* extracellular vesicles may also potentially enlarge our vision of REMORIN’s strictly PM function [37,38,39]. Concerning biotic stresses and in particular viruses, our teams were the pioneers in demonstrating that group 1 REM proteins are involved in plant–virus interactions, and in particular, that group 1 REM limit potexvirus propagation [40]. We also showed that REM’s phosphorylation status defines its PM nanodomain organisation and activity in restricting PVX cell-to-cell movement [32]. Since then, several studies about different REM groups showed that REMs may have agonist or antagonist effects on the infection by different viruses [30,41]. Some REMORIN members are indeed able to promote the viral propagation of two distinct virus genera. During rice stripe virus (RSV) infection, the RSV-encoded protein, NSvc4, targets the S-acylation of NbREM1.1 and 1.2 and promotes their autophagic degradation to facilitate viral spread [42].

Using a phage display screen to identify potential interactors of the potyvirus plum pox virus Cylindrical Inclusion, a 12-amino-acid peptide showing homology with *A. thaliana* group 4 REMORINs was identified. This prompted us to check whether an effect on viral propagation could apply to potyviruses. In the present study, we focused on the effect of StREM1.3 PM localisation and phosphorylation on two potyvirus species, TuMV and *Potato virus A* (PVA). We showed that StREM1.3 plays an agonistic effect on potyvirus propagation in *N. benthamiana*.

## 2. Materials and Methods

### 2.1. Plant Materials and Growth Conditions

Healthy and infected *N. benthamiana* plants were maintained in a separate insect-proof greenhouse compartment (18/25 °C night/day) or in a climate chamber (18/20 °C, 8 h night/16 h day). The generation of transgenic stable hairpin REM and 35S::GFP–REM1.3 *N. benthamiana* lines were previously described in [32]. The protein expression levels of endogenous NbREMs in the hpREM lines (hpREM1.4, hpREM2.1, hpREM10.2, and hpREM20.3) were determined by Western blot analysis using anti-REM [40]. Transgenic overexpressing StREM1.3 lines (#6, #7, and #16) were tested by Western blot against REM and showed that they contain at least three times the number of endogenous NbREMs [32].

### 2.2. Molecular Clones and Fluorescent Tagged Potyvirus Infectious Clones

The different molecular clones for the expression of fluorescent fusion proteins (RFP-StREM1.3, RFP-StREM1.3^1−170^, RFP-StREM1.3^AAA^) were described in [32,36]. The mCherry-PDCB1 PD marker (plasmodesmata callose-binding protein 1) was described in [43]. The pGreenTuMV/nGFP-cGUS infectious clone was kindly supplied by Dr. J.F. Laliberté. In this construct, both GUS and GFP genes were inserted in the TuMV-UK1 genome, flanked by viral protease recognition site sequences, leading to the production of free GUS or GFP fluorescent proteins during infection [44]. The pCambiaTuMV-6K2-mCherry, pCambiaTuMV/6K2:mCherry//GFP-HDEL, and the mutant pCambiaTuMV^W15A^/6K2:mCherry//GFP-HDEL constructs were kindly supplied by Dr. J. F. Laliberté [13]. The full-length infectious cDNA TuMV-UK1 is cloned into *Agrobacterium tumefaciens* binary vector under the control of a 35S transcription promoter and 35S terminator. In those constructs, the genome of TuMV is engineered to ectopically express the mCherry fluorescent protein fused at the C-terminus of the 6K2 (wild-type of the W15A-6K2 mutant form of 6K2). This allows the visualisation of the TuMV-produced 6K2:mCherry vesicles labelling the viral replication complex in the infected cells. The TuMV^W15A^ mutant is still replication-competent even though it does not reach the wild-type TuMV replication level, but it is not able to move from cell to cell [13]. The PVA–GFP construct was kindly supplied by Dr. K. Makinen [45]. This construct pKJE11 corresponds to a full-length infectious cDNA of PVA expressing a free GFP cloned between the NIb-and CP-coding regions. For agroinoculation of the viral constructs, *Agrobacterium tumefaciens* cells containing TuMV-derived clones and PVA–GFP construct were infiltrated into the *N. benthamiana* leaves at an OD_600_ of 0.007 and 0.005, respectively. Infection foci induced by TuMV–GFP and PVA–GFP were observed at 5 and 3 dpi, respectively, using the Microscope ZEISS AxioZoom. The surface area of the infectious foci was measured using ImageJ software.

### 2.3. GUS Assays

Histochemical assays for GUS (β-glucuronidase) activity were conducted following a protocol described in [46]. A vacuum was used to infiltrate the GUS staining solution supplemented with 0.2% (vol/vol) Triton X-100 in leaf fragments. Development of the colorimetric reaction was routinely performed overnight at 37 °C. The surface area of the infection foci induced by pGreenTuMV/nGFP-cGUS was estimated at 5 dpi using the Microscope ZEISS AxioZoom. The surface area of the infectious foci was measured using ImageJ software.

### 2.4. Transient Protein Expression and Confocal Microscopy Analyses

Four-week-old *N. benthamiana* plants for transient expression experiments using *Agrobacterium tumefaciens* were grown at 22–24 °C in a greenhouse. *A. tumefaciens* cells harbouring fusion proteins were cultured at 27 °C overnight to an OD_600_ of 0.8, to optimise protein expression. For coagroinfiltration and BiFC experiments, the *A. tumefaciens* suspensions were mixed in a 1:1 ratio. In this mixture, the final dilution of the agrobacteria expressing the fusion proteins was equivalent to an OD_600_ of 0.2. *Agrobacterium* mixtures were gently infiltrated with a 1 mL needle-free syringe in the lower epidermis of the *N. benthamiana* leaves. Three to five days after agroinoculation, leaf samples were imaged using confocal Zeiss LSM 880 microscopy with 20× and 40× or 63× oil immersion objectives to determine the subcellular localisation of the overexpressed proteins. Argon and DPSS lasers were used to excite fluorescent proteins, and signals from both green and red channels were collected simultaneously. GFP and YFP were excited at 488 nm, and the emission light was captured at 499 nm to 544 nm; mCherry was excited at 561 nm, and the emission light was captured at 579 nm to 633 nm. Image processing was performed with Image J.

### 2.5. Callose Quantification by Aniline Blue Staining and Colocalisation Analysis

To quantify the callose deposition induced by StREM1.3 and/or TuMV infection, 25 µg/mL of Alinine blue (Biosupplies, Australia) was infiltrated through the lower epidermal surface using a 1 mL needle-free syringe by gentle pressure, just before observation. The 405 nm diode laser was used to excite aniline blue fluorochrome, and emission light was captured between 463 nm and 491 nm. For the callose quantification experiment, all images were collected using the same parameters between the different tested conditions. This experiment was performed in biological triplicates. The fluorescence intensity of the aniline-blue-stained plasmodesmata was determined using manually drawn region of interest (ROI) on raw 8-bit images, from which was measured integrated density. Analysis was performed using the ImageJ software. Statistical analysis was performed using Student’s *t*-test.

The colocalisation between the YFP signal reconstituted in BiFC (nYFP–REM and CI–cYFP) and aniline blue labelling at the PD level was quantified using the centroid object-based method using the 3D objects counter plugin of ImageJ software [47]. The resolution limit of each image was determined with a numerical aperture of 1.4 for x63 oil objective and the shorter emission maximum wavelength of the fluorophores. When the distance between two labelled structures was below the resolution limit of the image (~220 nm), the colocalisation was considered as true and a % of colocalisation corresponding to the number of colocalising units on the total number of units was calculated. Statistical analysis was performed using the % of colocalisation value for at least 10 images, using a Kruskal–Wallis test.

### 2.6. RNA Extraction and RT–qPCR 

Total RNA extraction from *N. benthamiana* WT and stable transgenic lines infected with TuMV/6K2:mCherry//GFP-HDEL or TuMV^W15A^/6K2:mCherry//GFP-HDEL was performed using the NucleoSpin^®^ RNA Plant Kit (Macherey Nagel, SAS, Hoerdt, France). Five independent plants for each condition were sampled in three independent experiments. The purified RNAs were treated once again by Dnase I with the TURBO DNASE-free ^TM^ Kit (Invitrogen, Thermo Fisher Scientific, Waltham, MA, USA), and RNA concentration was determined by measuring absorbance at 230, 260, and 280 nm in a microplate UV–Vis spectrophotometer (Agilent BioTek Instrument, Fisher Scientific, Illkirch, France)). The total RNA was adjusted to 50 mg/mL and was reverse transcribed according to the manufacturer’s instructions using a RevertAid H Minus enzyme (Thermo Scientific, Waltham, MA, USA) and oligo (dT)(18) primer. cDNA was used to perform the real-time quantitative RT–PCR on the Light Cycler 480 Instrument II (Roche Diagnostics, Meylan, France), using the Light Cycler^®^480 SYBR Green I MASTER Kit (Roche). The PCR mixture included per well 10 µL of Master mix, 0.6 µL of each primer (0.3 mM) (Table 1), 3.8 µL of H_2_O, and 5 µL of cDNA. Thermal cycling conditions were as follows: 15 s at 95 °C, followed by 40 cycles of 5 s at 95 °C, 20 s at 57 °C, and 30 s at 72 °C. Viral levels were determined using a capsid primers pair CP1-F and CP2-R (Table 1) while the GFP-HDEL transcripts transcribed from the cassette of the same construct but independent of the TuMV virus were used as an internal control and amplified with GFP-HDEL_F and GFP-HDEL_R primers (Table 1).

We checked that the GFP-HDEL primers GFP-HDEL_F and GFP-HDEL_R (Table 1) do not amplify the GFP gene fused to REM in the #7 and #16 lines. The viral gene expression (CP) was normalised with the reference gene GFP-HDEL. The efficiencies (E) of PCR amplification for the target (CP) and the reference (GFP-HDEL) were calculated and considered in the calculation of mean normalised expression (MNE). Values are as log MNE, which allows the comparison between samples, as it normalises the expression with the GFP-HDEL reference gene [48]. [MNE = (Etarget)^Ct (target, mean)^/(Eref)^Ct (ref, mean)^] (E = 1.888 for the target CP and E = 1.954 for the GFP-HDEL target).

### 2.7. Interaction Analyses through Split-Ubiquitin Y2H and BiFC Assays

Split ubiquitin analysis was performed using the yeast two-hybrid system from the DUAL membrane system (Dualsystems Biotech AG, Schlieren, Switzerland). *StREM1.3* and *^TuMV^CI* cDNA were amplified by PCR using SfiI restriction site–containing primers (Table 1) with subsequent orientated cloning of *stREM1.3* cDNA into pBT3N bait and pPR3N prey vectors and *^TuMV^CI* cDNA into pBT3SUC bait vector. The constructs pBT3N:CER1 and pPR3N:CytB5-B were kindly supplied by Dr. A. Bernard [49] and were used as an internal positive control. THY.AP4 yeast strain (*MATa ura3 leu2 lexA-lacZ-TRP1 lexA-HIS3 lexA-ADE2*) cells were cotransformed with pBT3SUC:^TuMV^CI and pPR3N:StREM1.3. The positive control was the cotransformation with pBT3N:CER1 and pPR3N:CytB5-B constructs. The bait pBT3N:CER1 was used as a negative control for the prey pPR3N:StREM1.3. The prey pPR3N:CytB5-B was used as a negative control for the bait pBT3N:StREM1.3. Transformants were selected on TL medium plate, and interactions were assayed on HTL and AHTL medium plates supplemented with 2.5 mM 3-amino1,2,4-triazole (SIGMA).

Protein–protein interactions were assessed in planta by BiFC [50]. The cDNA encoding StREM1.3 was fused in-frame via LR Clonase Gateway reaction (Invitrogen) to the N-terminal half of YFP (NY) in the pSITE-nEYFP-C1 vector (ABRC CD3-1648) and ^TuMV^CI cDNA to the C-terminal half of YFP (CY) in the pSITE-cEYFP-N1 vector (ABRC CD3-1651). For fluorescence complementation tests, compatible combinations between protein pairs (i.e., providing both parts of the YFP) were assayed by transient expression following agroinfiltration of *N. benthamiana* leaves. YFP fluorescence was detected 3 days after infiltration by confocal fluorescence microscopy. The lambda scan mode confirmed that for some ROI spots, maximum fluorescence emission was observed at 530 nm, which corresponds to YFP emission wavelength.

### 2.8. Protein Analysis, Western Blots

Plant leaf tissue was ground in liquid nitrogen and 200 µL of the Laemmli buffer was added to the powder (200 mM Tris–HCl pH 6.8, 4% SDS, 20% glycerol, 0.05% bromophenol blue, and 100 mM DTT). Following boiling for 10 min, the homogenate was centrifuged at 10,000× *g* for 1 min. Proteins were separated on 10% polyacrylamide–SDS gels and blotted onto nitrocellulose membranes. The membranes were probed with antibodies raised against REM [40], and anti-rabbit IgGs coupled with peroxidase (Sigma-Aldrich, St Quentin Fallavier, France) were used as secondary antibodies. Detection was finally achieved using the ChemiDoc Imaging System (Bio-Rad, Marnes-la-Coquette, France) and the Amersham ECL Western Blotting Detection Kit.

### 2.9. Statistical Analysis

Statistical analysis was performed using R 4.1.2 software. As mentioned in the figure legends, statistical significance was performed determined using either a Kruskal–Wallis bilateral test or a one-way ANOVA, followed by a Student’s *t*-test.

## 3. Results

### 3.1. StREM1.3 Overexpression Increases Potyvirus Propagation in N. benthamiana

We previously showed that in tomato and *N. benthamiana*, StREM1.3 limits the cell-to-cell spread of the potexvirus PVX without affecting its replication [40]. Here, we investigated whether transient or stable overexpression of StREM1.3 would have a similar effect on the propagation of TuMV.

**Figure 1:**
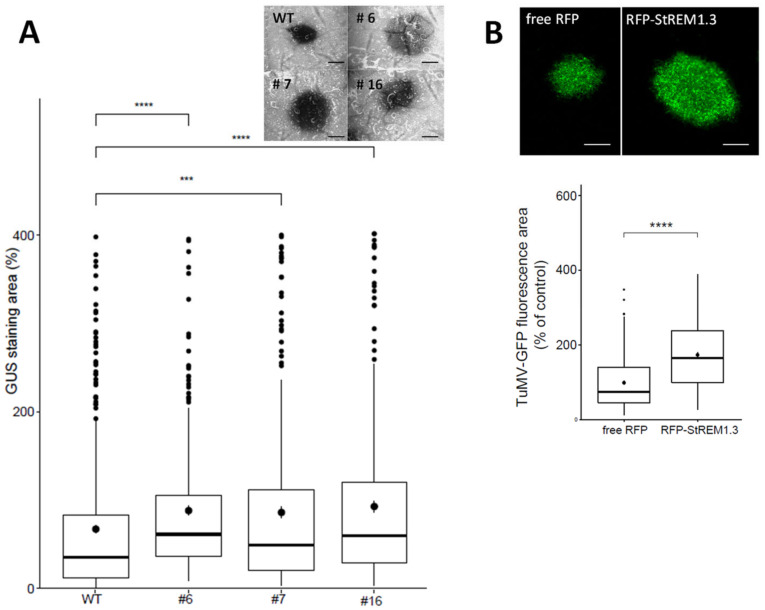
Stable and transient over-expression of StREM1.3 favour TuMV spreading in *N. benthamiana*: (**A**) effect of StREM1.3 overexpression in three independent stable transgenic *N. benthamiana* lines. The area of infection foci is normalised and expressed in % of the area of TuMV propagation in wild-type (WT) plants. Beta-glucuronidase tagging (GUS) of TuMV allowed the observation and measurement of infection surface foci induced by TuMV in the GFP–StREM1.3-overexpressing lines. At least 200 infection foci from at least 4 independent experimental repeats were imaged at 5 days post-inoculation (dpi). Statistical analysis was performed using one-way ANOVA, followed by Student’s *t*-test in R software (ANOVA, *p* = 0.0024; **** < 0.001, ***: 0.001). Scale bar: 500 µm; (**B**) effect of transient overexpression of RFP–StREM1.3. The area of infection foci is normalised and expressed in % of the area of virus propagation in the absence of REM (free RFP negative control). Statistical analysis was performed using one-way ANOVA, followed by Student’s *t*-test (ANOVA, *p* < 2.2 × 10^−16^; **** < 0.001). At least 200 infection foci from at least 4 independent repeats were imaged at 5 days post-inoculation (dpi). Scale bar: 500 µm.

Figure 1A shows that stable overexpression of GFP-StREM1.3 in three independent 35S::GFP–REM1.3 overexpressing transgenic lines leads to an increase in the surface of infection foci induced by TuMV in the inoculated leaves. Such a positive effect of stable REM overexpression was confirmed by transient overexpression of RFPStREM1.3 (Figure 1B) using free RFP as a negative control. The expression and subcellular localisation of StREM1.3 fused to RFP were examined in confocal microscopy. As expected, the RFP–StREM1.3 fusion protein discloses an association with the PM, while free RFP is nucleocytoplasmic (Figure S1A,B). This increase in the surface of TuMV infection foci was also confirmed with another potyviral species *Potato virus A* (PVA) (Figure S2A). In those experiments, as an internal control, we reproducibly observed the antagonistic effect on the potexvirus PVX–GFP propagation, as we published before [32,36]. Altogether, those results strongly suggest that StREM1.3 plays a proviral role in potyvirus infection in *N. benthamiana*.

### 3.2. The Propagation of TuMV Is Slowed Down in Lines Underexpressing Group 1 Endogenous NbREMs

To confirm the proviral role played by StREM1.3, we surveyed TuMV propagation in stably transformed *N. benthamiana* plants underexpressing group 1 endogenous NbREMs with a hairpin construct, as previously described in [32].

**Figure 2:**
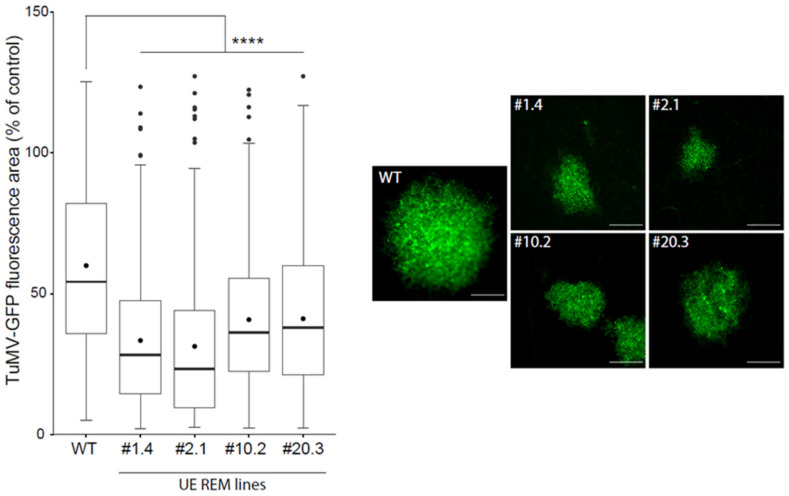
TuMV spreading is slowed down in four independent transgenic *N. benthamiana* lines underexpressing group 1 endogenous NbREMs (UE REM lines). The area of infection foci is normalised and expressed in % of the area of TuMV propagation in WT plants. GFP allowed the observation and measurement of infection surface foci induced by TuMV in the hairpin REM lines. At least 150 infection foci from at least 3 independent experiments were imaged at 5 days post-inoculation (dpi). Statistical analysis was performed using one-way ANOVA, followed by Student’s *t*-test in R software (ANOVA, *p* < 2.2 × 10^−16^; **** < 0.001). Scale bar: 500 µm.

Figure 2 shows that the TuMV infection foci surface in four independent stable transgenic *N. benthamiana* lines underexpressing group 1 NbREMs was significantly smaller than in WT plants. The same effect was observed for PVA–GFP propagation (Figure S2B). Our results, therefore, confirm that a positive correlation is observed between the expression of group 1 REMORINs and potyvirus propagation in the inoculated leaves of *N. benthamiana*.

### 3.3. StREM1.3 and NbREM Expression Levels Do Not Affect TuMV Replication

**Figure 3:**
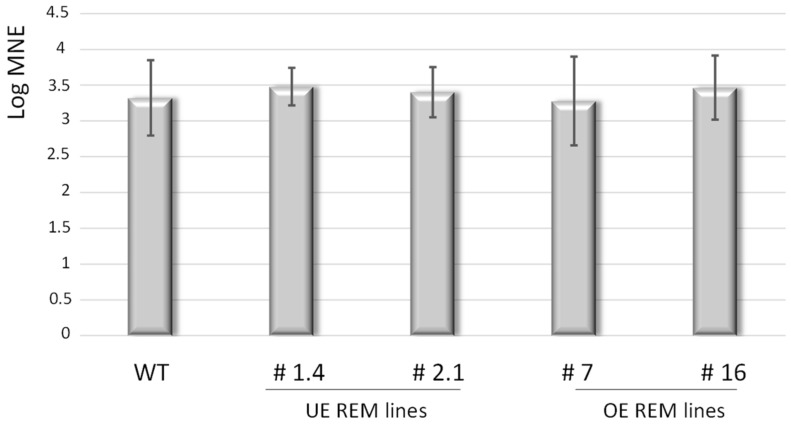
Relative CP gene expression of TuMV^w15A^ (log mean normalised expression, MNE) in the different *N. benthamiana* lines. TuMV CP gene expression was expressed as log mean normalised expression (MNE) in the different *N. benthamiana* genotypes at 5 dpi. The CP gene expression was normalised with the reference gene *GFP-HDEL*. Gene expression was quantified in the inoculated leaves sampled from 5 independent plants in three independent experiments. Three technical replicates were performed for each sample. A Kruskal–Wallis test was performed. No significant difference was observed between the different genotypes (alpha risk = 0.05). WT: *N. benthamiana* wild type. UE REM lines: *N. benthamiana* lines underexpressing group 1 endogenous NbREMs. OE REM lines: 35S::GFP–REM1.3 overexpressing transgenic lines.

The modified spread of TuMV in overexpressing or hairpin lines might be explained by reduced virus replication. To assess the accumulation level of TuMV independently of the movement step in the different lines (WT, over- and underexpressing REM lines), we used the TuMV^W15A^/6K2:mCherry//GFP-HDEL construct. This expression clone contains in the same cassette a GFP-tagged HDEL and a TuMV sequence with a mCherry-tagged 6K2 that is mutated on its tryptophan 15. This mutation totally impedes cell-to-cell propagation, while it slightly affects viral replication [13]. We first controlled that this mutant was able to replicate in the different genotypes and observed an increase in viral accumulation during a preliminary time-course experiment in WT, line #16, and hpREM1.4. We then compared the accumulation of TuMV^W15A^ in WT and stable transgenic lines overexpressing StREM1.3 (lines #7 and #16) or underexpressing NbREMs (‘hairpin lines’, hp1.4 and hp2.1). TuMV^W15A^ replication levels in the infected cells were determined by quantitative real-time PCR, using a TuMV–capsid primers pair (CP) and normalised with the reference gene GFP-HDEL. As shown in Figure 3, the level of accumulation of TuMV^w15A^ at 5 dpi is equivalent in all the *N. benthamiana* lines overexpressing or silenced for REM. Therefore, the level of expression of REM does not impact the level of replication of TuMV but its cell-to-cell movement.

### 3.4. StREM1.3 Membrane Anchoring Is Necessary for Increasing Potyvirus Cell-to-Cell Movement

**Figure 4:**
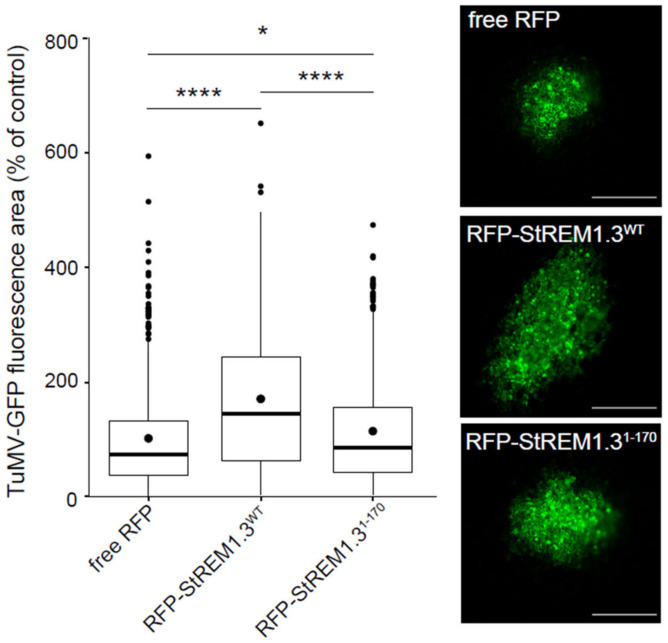
REM–CA-mediated PM binding is required for StREM1.3 increasing TuMV cell-to-cell propagation. Effect of transient overexpression of RFP-StREM1.3^1–170^. The area of infection foci is normalised and expressed in % of the area of virus propagation in the absence of REM (RFP negative control). At least 350 infection foci from at least 4 independent experiments were imaged at 5 days post-inoculation (dpi). Statistical analysis was performed using one-way ANOVA, followed by Student’s *t*-test in R software (ANOVA *p* < 2.2 × 10^−16^; **** < 0.001/*: 0.05). Scale bar: 500 µm.

We previously showed that a C-terminal anchor peptide 170–198 from StREM1.3, called REM–CA, is required for a strong PM anchoring [36,51,52]. Deletion of the REM–CA resulted in an entire loss of PM association of the corresponding mutant StREM1.3^1–170^ and abolished StREM1.3 function in restricting PVX movement. Here, we examined whether transient overexpression of StREM1.3^1–170^ would still have a positive effect on TuMV propagation. Figure S1C confirms the major loss of PM localisation for this mutant in *N benthamiana*. Figure 4 shows that overexpression of RFP-StREM1.3^1–170^ has barely any effect on TuMV propagation, compared with the strong effect observed with RFP–StREM1.3^WT^.

Similar results were obtained with PVA–GFP (Figure S2A). In conclusion, the agonistic effect on potyvirus propagation is severely impaired by the removal of the REM–CA domain suggesting that the anchoring of StREM1.3 to the PM is necessary for the positive effect on potyvirus movement.

### 3.5. StREM1.3 Phosphorylation Is Involved in Increasing Potyvirus Cell-to-Cell Movement

**Figure 5:**
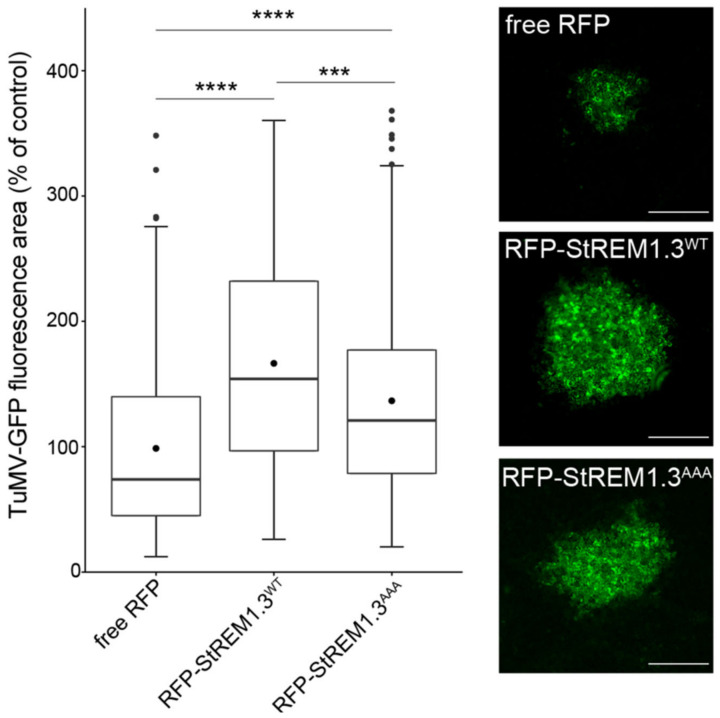
The phosphodead mutant StREM1.3^AAA^ still increases TuMV cell-to-cell movement but less than WT–StREM1.3. Effect of transient overexpression of RFP–StREM1.3^AAA^. The area of infection foci is normalised and expressed in % of the area of virus propagation in the absence of REM (free RFP negative control). At least 200 infection foci from at least 4 independent experiments were imaged at 5 days post-inoculation (dpi). Statistical analysis was performed using one-way ANOVA, followed by Student’s *t*-test in R software (ANOVA, *p* < 2.2 × 10^−16^; **** < 0.001, ***: 0.001). Scale bar: 500 µm.

We previously showed that StREM1.3’s phosphorylation status seems crucial for StREM1.3-dependent restriction of PVX cell-to-cell movement [32]. Here, we investigated whether the phosphodead mutant StREM1.3^AAA^ (corresponding to the triple mutation S74A/T86A/S91A) was still able to improve potyvirus propagation. Transient expression of RFP–StREM1.3^AAA^ in *N. benthamiana* coupled with TuMV–GFP infection assays demonstrate that the triple mutant only induces a partial loss of function of StREM1.3 in increasing TuMV–GFP (Figure 5) or PVA–GFP propagation (Figure S2A).

This result suggests that the phosphocode previously described as involved in the inhibition of PVX propagation is not fully at play during potyvirus propagation.

### 3.6. TuMV Infection Does Not Alter the Expression Level of Endogenous NbREM, Nor the Membranous Localisation of GFP–StREM1.3

**Figure 6:**
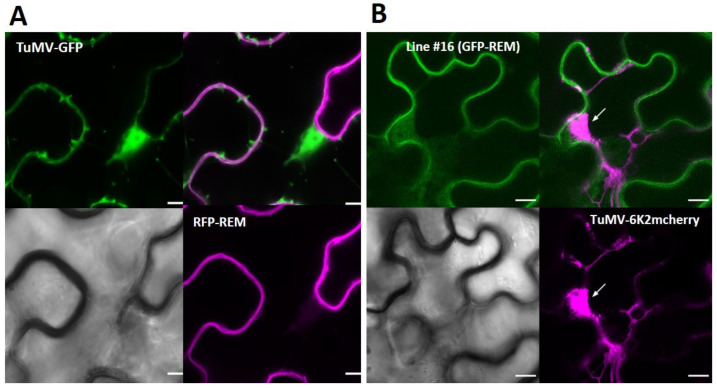
TuMV infection does not modify StREM1.3 plasma membrane localisation: (**A**) transient expression of RFP–StREM1.3. Representative confocal images showing the membranous transiently expressed RFP–StREM1.3 in *N. benthamiana* leaf epidermal cells infected by TuMV–GFP at 5 dpi. RFP–StREM1.3 remains membranous; (**B**) stable overexpression of GFP–StREM1.3. Representative confocal images showing the membranous expressed GFP–StREM1.3 in the overexpressing transgenic line #16 during TuMV-6K2mcherry infection at 5 dpi. The red signal labels the 6K2-induced vesicles and the viral replication complex (VRC) near the nucleus as indicated by the white arrow. Scale bar = 10 µm.

To investigate whether TuMV infection alters the level of endogenous NbREMs, we performed a kinetic analysis of TuMV infection, followed by a Western blot analysis of the level of NbREMs. The TuMV–GFP construct was agroinoculated, and the negative control “mock” corresponded to free GFP expressed alone. Figure S3 shows that the level of NbREM is not affected by the viral infection in the inoculated leaves at the different time points of the kinetics. To investigate any potential effect of TuMV infection on REM subcellular localisation, TuMV–GFP and TuMV-6K2mcherry viruses were agroinoculated in *N. benthamiana* leaves, where RFP–StREM1.3 was transiently expressed and in the transgenic line #16 overexpressing GFP–StREM1.3, respectively. TuMV–GFP infection allows the visualisation of a green signal corresponding to the nucleocytoplasmic GFP expressed from the virus during infection. The RFP–StREM1.3 fusion protein remains located at the PM at 5 dpi in TuMV–GFP-inoculated leaves (Figure 6A) and also in PVA-infected leaves (Figure S4).

TuMV-6K2mcherry infection allows the visualisation of a mcherry fluorescent protein fused at the C-terminus of the membranous viral protein 6K2 that labels the viral replication complex near the nucleus. The GFP–StREM1.3 expressed in transgenic line #16 is still located in the PM after TuMV-6K2mcherry infection at 5 dpi in the inoculated leaves (Figure 6B). Figure 6B clearly shows that even during TuMV-6K2mcherry infection, GFP–StREM1.3 localisation remains at the PM. The expected patchy distribution can also be observed at the surface view of the cell in different focal planes. The very faint signal overlap of GFP and mcherry signals is not representative of the majority of our observations. Therefore, both TuMV and PVA infections do not modify StREM1.3 plasma membrane localisation.

### 3.7. StREM1.3 Interacts in Yeast and in Planta with ^TuMV^CI, a Viral Protein Crucial for Potyvirus Movement

**Figure 7:**
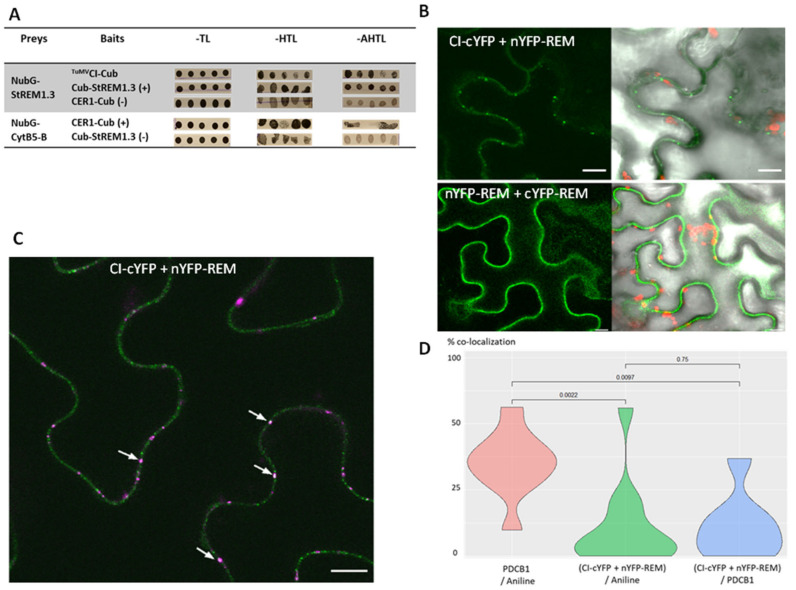
StREM1.3 interacts in yeast and in planta with ^TuMV^CI: (**A**) interaction between StREM1.3 and ^TuMV^CI in yeast split-ubiquitin assays. Five independent yeast colonies cotransformed with NubG–StREM1.3 and ^TuMV^CI-Cub resulted in vigorous growth on synthetic dropout (SD) medium lacking Leu and Trp (-TL). Interaction is indicated by yeast growth on SD medium containing 2.5 mM 3-AT (3-amino-1,2,4-triazole) and lacking Leu, Trp, His, and Ade (-AHTL). Bait Cub–StREM1.3 (cloned in pBT3N): Cub moiety fused at the N-ter of StREM1.3; prey StREM1.3 (cloned in pPR3N): NubG moiety fused at the N-ter of the protein. Prey ^TuMV^CI (cloned in pBT3SUC): Cub moiety fused at the C-ter of ^TuMV^CI. Positive controls: CER1- CytB5b and StREM1.3 autointeraction. Negative controls: StREM1.3 with CER1 or with CytB5b; (**B**) bimolecular fluorescence complementation (BiFC) detection of interactions between nYFP–REM and CI–cYFP in *N. benthamiana* epidermal cells. Images were captured using a confocal microscope at 3 days post-infiltration (dpi). Images are single confocal sections. Positive control: autointeraction of StREM1.3 (nYFP–REM + cYFP–REM). Scale bar: 10 µm; (**C**) representative confocal image showing aniline blue staining of callose deposition at the PD pit fields in *N. benthamiana* leaf cells expressing nYFP–REM and CI–cYFP. Callose deposition is labelled in pink. White arrows indicate colabelled spots in the PM corresponding to the YFP (BiFC signal) and aniline blue signals. Scale bar: 10 µm; (**D**) partial colocalisation between PD pit fields and YFP signal reconstituted in BiFC (nYFP–REM and CI–cYFP). The percentage of colabelled spots per image is indicated for each combination: colocalisation between PD marker PDCB1 and aniline blue, YFP signal (reconstituted after StREM1.3 and ^TuMV^CI interaction), and aniline blue or PDCB1. At least 10 images per condition were analysed. Statistical analysis was performed using Kruskal–Wallis test in R software.

Among the potyviral proteins, the cylindrical inclusion (CI) is the component of the characteristic pinwheel-shaped inclusion bodies in the cytoplasm of infected cells, a unique feature shared by all *Potyviridae*. CI forms cylindrical inclusions (hence its name) in the cytoplasm adjacent to PD in infected cells and is associated with cone-shaped structures close to PD, together with CP and viral RNA [8,53]. As we showed that StREM1.3 favours TuMV propagation, we examined whether StREM1.3 protein could directly interact with ^TuMV^CI. To assess this interaction, split-Ubiquitin Y2H assays were conducted. The results show that NubG–StREM1.3 interacts with ^TuMV^CI-Cub (Figure 7A). Two positive controls were performed: the autointeraction of StREM1.3 and the interaction between CER1 (a protein essential for wax alkane synthesis) and its cofactor CYTB5 [49].

We used the lack of interaction between StREM1.3 with CER1 or CYTB5 as negative controls. To confirm that StREM1.3 interacts with ^TuMV^CI, we performed bimolecular fluorescence complementation (BiFC) assays in planta. Different combinations of StREM1.3 and ^TuMV^CI transient expression vectors were agroinfiltrated in *N. benthamiana* to test whether the proteins could interact. Among those combinations, some of them did not lead to any signal. Those combinations are cYFP–REM + CI–nYFP, cYFP–REM + nYFP–CI, and nYFP–REM + cYFP–CI. However, as shown in Figure 7B, fluorescent signals are observed as pit fields on the PM of *N. benthamiana* epidermal cells coexpressing either nYFP–REM and CI–cYFP or the positive control cYFP–REM and nYFP–REM, whereas no signal is detected in the negative controls performed (i.e., only nYFP–REM or CI–cYFP expressed alone or the negative combinations listed above). The lambda scan mode confirmed that for some ROI spots, maximum fluorescence emission was observed at 530 nm, which corresponds to YFP emission wavelength (Figure S5). Aniline blue staining of callose (β-1,3-glucan), a structural and functional component of plasmodesmata (PD), showed that colabelling is observed between aniline blue staining and nYFP–REM and CI–cYFP interaction (Figure 7C). Compared with the situation in which a PD marker protein (PDCB1) is coexpressed with aniline blue staining, the percentage of colabelled spots in the nYFP–REM and CI–cYFP interaction condition is significantly lower than the percentage observed with PDCB1 (Figure 7D and Figure S6). Altogether, these results show that StREM1.3 interacts directly with ^TuMV^CI protein at the PM level revealing a patchy pattern of interaction, although not specifically at the PD level.

### 3.8. Callose Deposition Induced by StREM1.3 Is Decreased by TuMV Infection

**Figure 8:**
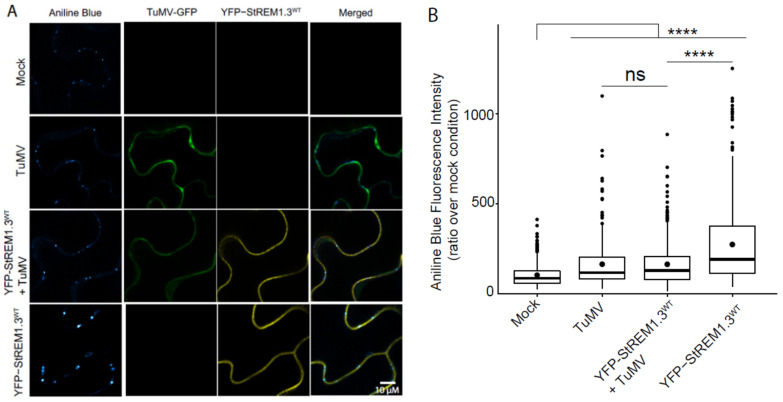
TuMV prevents StREM1.3-induced callose accumulation at plasmodesmata: (**A**) representative confocal images of aniline blue-stained *N. benthamiana* leaf epidermis cells expressing either YFP–StREM1.3, TuMV–GFP, or both. Colour code of the aniline blue track indicates intensity (the lightest the colour, the more intense). Mock corresponds to the negative control (empty vector agrobacteria); (**B**) quantification of callose accumulation at plasmodesmata was performed by measuring aniline blue fluorescence at pit field using ImageJ, as previously described in Perraki et al. (2018). At least 392 PD pi -fields from 3 independent experiments were imaged at 3 days post-inoculation (dpi). Statistical analysis was performed using one-way ANOVA, followed by Student’s *t*-test in R software (ANOVA, *p* < 2.2 × 10^−16^; **** < 0.001). Scale bar: 10 µm.

The REM-dependent restriction of PVX cell-to-cell movement was shown to be linked to the regulation of callose deposition at PD [32]. In this study, we quantified callose deposition in TuMV–GFP-infected cells. Our results (Figure 8) confirm our previous data showing that RFP–StREM1.3 induces an increase in callose deposition at PD. We also found that RFP–StREM1.3 induces an increase in callose deposition at PD in the infected leaves, compared with the mock condition (Figure 8A), and that this effect is antagonised by TuMV infection (Figure 8B). Therefore, TuMV infection has a negative impact on the callose deposition induced by StREM1.3.

## 4. Discussion

### 4.1. REMORIN Proteins Have Antagonistic or Agonistic Roles in Viral Cell-to-Cell Movement 

Our teams were pioneers in demonstrating that group 1 REMs of Solanaceae plants (StREM1.3, NbREM1.2, and NbREM1.3) impair the cell-to-cell movement of the potexvirus species PVX [32,35]. We confirmed this result for another potexvirus plantago asiatica mosaic virus (PlAMV) (unpublished results) and for a tobamovirus species (TMV) [32]. Since then, overexpression of NbREM1.1/1.2 or rice OsREM1.4 was shown to also impair the cell-to-cell movement of the tenuivirus rice stripe virus (RSV) [42]. *Arabidopsis thaliana* REMORIN proteins in group 4 were reported to be positive regulators for infections by geminiviruses [41]. Furthermore, overexpression of StREM1.3 limits TMV propagation in *N. benthamiana*, but transient expression of NtREM1.2 (phylogenetically close to StREM1.3) favours the propagation of another tobamovirus species *Tomato mosaic virus* (ToMV) in the same host [54].

Cheng et al. (2020) described how overexpression of Arabidopsis AtREM1.2 in *N. benthamiana* negatively regulates the cell-to-cell movement of TuMV [29]. This last result appears at variance with our data, where we showed that overexpression of StREM1.3 in *N. benthamiana* favours the cell-to-cell movement of two potyviral species, TuMV and PVA while impairing PVX propagation in the same set of experiments. It must be pointed out that the same TuMV isolate, UK1, was used in Cheng’s study and ours, although not the exact same molecular construct used for agroinoculation assays. We can hypothesise that gain of function with the overexpression of StREM1.3 or AtREM1.2, although belonging to the same group 1 of REMORINS [30], has different effects on potyvirus when overexpressed in *N. benthamiana*. Such a hypothesis does not apply to potexviruses, as overexpression of AtREM1.2 or StREM1.3 both restricts PVX–GFP cell-to-cell movement in *N. benthamiana*. [32]. Importantly, in our study, loss of function of group 1 NbREM in *N. benthamiana* lead to a decrease in TuMV and PVA propagation. Altogether, these results suggest that REMORINs of different groups may have different roles in controlling virus susceptibility depending on the virus species and also that group 1 REMORINs can act as pro- or antiviral factors in *N. benthamiana*, adding another level of complexity concerning the functions of REMORINs in viral infections. 

To date, all the studies describing REMORINs’ effects on viral infection, including our present study, showed that group 1 REMORIN protein levels affect the cell-to-cell movement of the virus but not its replication [29,35,40,42].

Using the StREM1.3^1–170^ mutant, we also revealed that the positive effect on the cell-to-cell movement of TuMV and PVA relies on StREM1.3 membrane anchoring, confirming our previous results showing that StREM1.3 PM binding is required for restriction of PVX cell-to-cell propagation likely by regulating signalling events at the PM [36]. Our results with the phosphodead mutant StREM1.3^AAA^ revealed that the phosphorylation at the three amino acid positions crucial for the antagonistic effect on PVX [32] is not crucial for potyvirus. We may hypothesise that a phosphocode exists and that phosphorylation is also involved in the TuMV agonist effect but involves other phosphoresidues. This remains to be determined.

### 4.2. TuMV Infection in N. benthamiana Does Not Affect Neither the Levels of Endogenous REMORIN Proteins, Nor the Plasma Membrane Localisation of Fluorescently Tagged StREM1.3

By contrast with the results of [42], which showed that NbREM1 is downregulated during RSV infection in *N. benthamiana*, we showed here that the level of endogenous NbREMS was not modified by TuMV infection, as described during PVX infection [36]. Although in one study [29], the authors showed that in *N. benthamiana*, transiently expressed HA-AtREM1.2 is degraded during TuMV infection ([28] Figure S10), there is no indication concerning the effect of TuMV infection on the endogenous NbREMs protein levels.

In our study, both transient and stable expression experiments using fluorescently tagged StREM1.3 protein showed that the patchy PM-associated localisation of StREM1.3 was not altered during infection by the two potyviral species TuMV and PVA. Our results differ from what was observed by [54], who showed that ToMV infection promoted the aggregation of NtREM-DsRed at sites close to cortical ERs in *N. benthamiana*. Using higher-resolution microscopy (spt-PALM), we had previously shown that StREM1.3 dynamic localisation at the PM was modified upon PVX infection [32]. Without performing such high-resolution microscopy observations, we, therefore, cannot exclude in our present study such an impact of potyvirus infection on StREM1.3 in the PD pit field regions.

### 4.3. TuMV Infection Interferes with the StREM1.3-Mediated Callose Deposition in N. benthamiana

We previously showed that overexpression of StREM1.3 limits the passage of macromolecules such as free GFP through the PD [55]. The PD conductivity can be disrupted by the callose (a β-1-3 glucan polymer) deposition at the neck region of PD, which strongly limits the diffusion of macromolecules through the pores [56]. StREM1.3 may have a direct function on PD conductivity, as StREM1.3’s localisation at the PM nanodomains and its phosphorylation status regulate callose deposition at the neck regions of PD [32,35,55]. We also previously showed that StREM1.3 affects the plasmodesmal gating activity of movement proteins (MP) such as the TRIPLE-GENE BLOCK protein 1 (TGBp1) from PVX, 30K from TMV, and also HC-Pro, a multifunctional helper protein of the potyvirus potato virus Y (PVY) [55]. In our present study, we showed that the callose deposition induced by StREM1.3 at the level of PD is decreased by TuMV infection. The underlying mechanisms are still not known for potyviruses, but we can hypothesise that this reduction could be either due to the inhibition of callose synthase or activation of β-glucanases or to the suppression of stress-associated synthesis of callose mediated by StREM1.3 at the PD during potyviral infection as suggested by [57].

### 4.4. The Interaction between ^TuMV^CI and StREM1.3 Occurs at the PM but Is Not Predominantly Associated to PDs: Could This Interaction Play a Role in the Non-PD Movement of Potyviruses?

The cylindrical inclusion (CI) of TuMV is a multifunctional scaffolding protein crucial for potyvirus movement [5] and is the component of the characteristic pinwheel-shaped inclusion bodies, a unique feature shared by all *Potyviridae* [8]. Here, we showed that ^TuMV^CI interacts with StREM1.3 in SuY2H, an interaction that was not observed with AtREM1.2 by [29] using the same SuY2H assays ([28] Figure S11), highlighting another discrepancy between AtREM1.2 and StREM1.3 properties. In our study, the interaction between ^TuMV^CI and StREM1.3 occurs at the level of PM but is not predominantly associated with PD. A recent change in paradigm for potyvirus movement was recently proposed by the authors of [18], who showed that TuMV components (including CI) could be released in the extracellular space of infected *N. benthamiana* within extracellular vesicles. These extracellular vesicles of TuMV could move in paramural space, as was suggested before in [58]. As group 1 REMORINs were identified in the extravesicular proteome [38,39], we can also hypothesise that REM would play another role in potyvirus movement at the PM by helping the exocytosis of TuMV-induced vesicles. 

Our present study shows another level of specificity and the complexity of the mode of action of REMORIN proteins during plant virus infection. We are only starting to identify the mechanisms, leading to an increase in TuMV and PVA propagation in the presence of StREM1.3, and further study of the REM phosphocode and its interaction with potyviral proteins could help unravel the mechanisms behind its positive effect on viral propagation.

## Figures and Tables

**Table 1:** List of primers used in this study.

Primer Name	Sequence (5′–3′)
CP1-F	TCAAACCATGTGTCAAACTCG
CP2-R	CGAGAAAAGAGTGGCCCTAA
GFP-HDEL_F	CCTGTCCTTTTACCAGAC
GFP-HDEL_R	CTCGTCATGAGATCTGTATAG
^TuMV^CI-SfiI-F	ATTAACAAGGCCATTACGGCCATGACTCTCAATGATATAGAGGA
^TuMV^CI-SfiI-R	AACTGATTGGCCGAGGCGGCCCCCTATTGATGGTGAACTGCCTC
St-REM1.3-SfiI-F	ATTAACAAGGCCATTACGGCCATGGCAGAATTGGAAGCTAAGAAAG
St-REM1.3-SfiI-R	AACTGATTGGCCGAGGCGGCCTCAAAATATTCCAAGGATTTTCTTTGG

The sequence of SfiI site is underlined.

## Data Availability

The datasets used and/or analysed during the current study are available from the corresponding author on reasonable request.

## Supplementary Materials

The following supporting information can be downloaded at: https://www.mdpi.com/article/10.3390/v14030574/s1, Figure S1: Confocal microscopy images representative of the subcellular localisation of transiently expressed proteins 3 days after agroinfiltration, Figure S2: (A) Effect of transient overexpression of stREM1.3 and its mutant forms on PVA spreading in *N. benthamiana*; (B) PVA spreading is slowed down in four independent transgenic *N. benthamiana* lines constitutively expressing hairpin REM constructs, Figure S3: The levels of endogenous *N. benthamiana* REMs are not modified by TuMV–GFP infection, Figure S4: PVA infection does not modify StREM1.3 membranous localisation, Figure S5: Use of the Lambda scan mode to confirm the nature of fluorescence emission signal in BiFC experiments, Figure S6: Representative confocal image showing aniline blue staining of callose deposition at the PD pit fields in *N. benthamiana* leaf cells expressing mcherry-PDCB1. Video S1, Z-Stack of confocal images showing the membranous transiently expressed RFP–StREM1.3 in *N. benthamiana* leaf epidermal cells infected by TuMV–GFP at 5 dpi.

## References

[1] Reagan B.C., Burch-Smith T.M. (2020). Viruses Reveal the Secrets of Plasmodesmal Cell Biology. Mol. Plant-Microbe Interact..

[2] Schoelz J.E., Harries P.A., Nelson R.S. (2011). Intracellular Transport of Plant Viruses: Finding the Door out of the Cell. Mol. Plant.

[3] Heinlein M., Heinlein M. (2015). Plasmodesmata: Channels for Viruses on the Move. Plasmodesmata.

[4] Wylie S.J., Adams M., Chalam C., Kreuze J., López-Moya J.J., Ohshima K., Praveen S., Rabenstein F., Stenger D., Wang A. (2017). ICTV Virus Taxonomy Profile: Potyviridae. J. Gen. Virol..

[5] Wang A. (2021). Cell-to-Cell Movement of Plant Viruses via Plasmodesmata: A Current Perspective on Potyviruses. Curr. Opin. Virol..

[6] Deng P., Wu Z., Wang A. (2015). The Multifunctional Protein CI of Potyviruses Plays Interlinked and Distinct Roles in Viral Genome Replication and Intercellular Movement. Virol. J..

[7] Movahed N., Patarroyo C., Sun J., Vali H., Laliberté J.-F., Zheng H. (2017). Cylindrical Inclusion Protein of Turnip Mosaic Virus Serves as a Docking Point for the Intercellular Movement of Viral Replication Vesicles. Plant Physiol..

[8] Sorel M., Garcia J.A., German-Retana S. (2014). The *Potyviridae* Cylindrical Inclusion Helicase: A Key Multipartner and Multifunctional Protein. Mol. Plant-Microbe Interact..

[9] Wei T., Zhang C., Hong J., Xiong R., Kasschau K.D., Zhou X., Carrington J.C., Wang A. (2010). Formation of Complexes at Plasmodesmata for Potyvirus Intercellular Movement Is Mediated by the Viral Protein P3N-PIPO. PLoS Pathog..

[10] Beauchemin C., Boutet N., Laliberte J.F. (2007). Visualization of the Interaction between the Precursors of VPg, the Viral Protein Linked to the Genome of Turnip Mosaic Virus, and the Translation Eukaryotic Initiation Factor Iso 4E in Planta. J. Virol..

[11] Cotton S., Grangeon R., Thivierge K., Mathieu I., Ide C., Wei T., Wang A., Laliberté J.-F. (2009). Turnip Mosaic Virus RNA Replication Complex Vesicles Are Mobile, Align with Microfilaments, and Are Each Derived from a Single Viral Genome. J. Virol..

[12] Grangeon R., Jiang J., Wan J., Agbeci M., Zheng H., Laliberté J.-F. (2013). 6K2-Induced Vesicles Can Move Cell to Cell during Turnip Mosaic Virus Infection. Front. Microbiol..

[13] Jiang J., Patarroyo C., Garcia Cabanillas D., Zheng H., Laliberté J.-F. (2015). The Vesicle-Forming 6K _2_ Protein of Turnip Mosaic Virus Interacts with the COPII Coatomer Sec24a for Viral Systemic Infection. J. Virol..

[14] Schaad M.C., Jensen P.E., Carrington J.C. (1997). Formation of Plant RNA Virus Replication Complexes on Membranes: Role of an Endoplasmic Reticulum-Targeted Viral Protein. EMBO J..

[15] Chai M., Wu X., Liu J., Fang Y., Luan Y., Cui X., Zhou X., Wang A., Cheng X. (2020). P3N-PIPO Interacts with P3 via the Shared N-Terminal Domain To Recruit Viral Replication Vesicles for Cell-to-Cell Movement. J. Virol..

[16] Den Boon J.A., Diaz A., Ahlquist P. (2010). Cytoplasmic Viral Replication Complexes. Cell Host Microbe.

[17] Cabanillas D.G., Jiang J., Movahed N., Germain H., Yamaji Y., Zheng H., Laliberté J.-F. (2018). Turnip Mosaic Virus Uses the SNARE Protein VTI11 in an Unconventional Route for Replication Vesicle Trafficking. Plant Cell.

[18] Movahed N., Cabanillas D.G., Wan J., Vali H., Laliberté J.-F., Zheng H. (2019). Turnip Mosaic Virus Components Are Released into the Extracellular Space by Vesicles in Infected Leaves. Plant Physiol..

[19] Vijayapalani P., Maeshima M., Nagasaki-Takekuchi N., Miller W.A. (2012). Interaction of the Trans-Frame Potyvirus Protein P3N-PIPO with Host Protein PCaP1 Facilitates Potyvirus Movement. PLoS Pathog..

[20] Park S.-H., Li F., Renaud J., Shen W., Li Y., Guo L., Cui H., Sumarah M., Wang A. (2017). NbEXPA1, an α-Expansin, Is Plasmodesmata-Specific and a Novel Host Factor for Potyviral Infection. Plant J..

[21] Hofius D., Maier A.T., Dietrich C., Jungkunz I., Bornke F., Maiss E., Sonnewald U. (2007). Capsid Protein-Mediated Recruitment of Host DnaJ-Like Proteins Is Required for Potato Virus Y Infection in Tobacco Plants. J. Virol..

[22] Cui X., Lu L., Wang Y., Yuan X., Chen X. (2018). The Interaction of Soybean Reticulon Homology Domain Protein (GmRHP) with Soybean Mosaic Virus Encoded P3 Contributes to the Viral Infection. Biochem. Biophys. Res. Commun..

[23] Geng C., Cong Q.-Q., Li X.-D., Mou A.-L., Gao R., Liu J.-L., Tian Y.-P. (2015). Developmentally regulated plasma membrane protein of *Nicotiana benthamiana* Contributes to Potyvirus Movement and Transports to Plasmodesmata via the Early Secretory Pathway and the Actomyosin System. Plant Physiol..

[24] Movahed N., Sun J., Vali H., Laliberté J.-F., Zheng H. (2019). A Host ER Fusogen Is Recruited by *Turnip Mosaic Virus* for Maturation of Viral Replication Vesicles. Plant Physiol..

[25] Uchiyama A., Shimada-Beltran H., Levy A., Zheng J.Y., Javia P.A., Lazarowitz S.G. (2014). The Arabidopsis Synaptotagmin SYTA Regulates the Cell-to-Cell Movement of Diverse Plant Viruses. Front. Plant Sci..

[26] Wu G., Cui X., Chen H., Renaud J.B., Yu K., Chen X., Wang A. (2018). Dynamin-Like Proteins of Endocytosis in Plants Are Coopted by Potyviruses To Enhance Virus Infection. J. Virol..

[27] Wu G., Cui X., Dai Z., He R., Li Y., Yu K., Bernards M., Chen X., Wang A. (2020). A Plant RNA Virus Hijacks Endocytic Proteins to Establish Its Infection in Plants. Plant J..

[28] Wu G., Jia Z., Ding K., Zheng H., Lu Y., Lin L., Peng J., Rao S., Wang A., Chen J. (2022). Turnip Mosaic Virus Co-Opts the Vacuolar Sorting Receptor VSR4 to Promote Viral Genome Replication in Plants by Targeting Viral Replication Vesicles to the Endosome. PLoS Pathog..

[29] Cheng G., Yang Z., Zhang H., Zhang J., Xu J. (2020). Remorin Interacting with PCaP1 Impairs *Turnip Mosaic Virus* Intercellular Movement but Is Antagonised by VPg. New Phytol..

[30] Gouguet P., Gronnier J., Legrand A., Perraki A., Jolivet M.-D., Deroubaix A.-F., German-Retana S., Boudsocq M., Habenstein B., Mongrand S. (2020). Connecting the Dots: From Nanodomains to Physiological Functions of REMORINs. Plant Physiol..

[31] Raffaele S., Mongrand S., Gamas P., Niebel A., Ott T. (2007). Genome-Wide Annotation of Remorins, a Plant-Specific Protein Family: Evolutionary and Functional Perspectives. Plant Physiol..

[32] Perraki A., Gronnier J., Gouguet P., Boudsocq M., Deroubaix A.-F., Simon V., German-Retana S., Legrand A., Habenstein B., Zipfel C. (2018). REM1.3’s Phospho-Status Defines Its Plasma Membrane Nanodomain Organization and Activity in Restricting PVX Cell-to-Cell Movement. PLoS Pathog..

[33] Raffaele S., Bayer E., Mongrand S. (2009). Upregulation of the Plant Protein Remorin Correlates with Dehiscence and Cell Maturation: A Link with the Maturation of Plasmodesmata?. Plant Signal. Behav..

[34] Marín M., Ott T. (2012). Phosphorylation of Intrinsically Disordered Regions in Remorin Proteins. Front. Plant Sci..

[35] Gronnier J., Crowet J.-M., Habenstein B., Nasir M.N., Bayle V., Hosy E., Platre M.P., Gouguet P., Raffaele S., Martinez D. (2017). Structural Basis for Plant Plasma Membrane Protein Dynamics and Organization into Functional Nanodomains. eLife.

[36] Perraki A., Cacas J.L., Crowet J.M., Lins L., Castroviejo M., German-Retana S., Mongrand S., Raffaele S. (2012). Plasma Membrane Localization of Solanum Tuberosum Remorin from Group 1, Homolog 3 Is Mediated by Conformational Changes in a Novel C-Terminal Anchor and Required for the Restriction of Potato Virus X Movement. Plant Physiol..

[37] Tran T.M., Chng C.-P., Pu X., Ma Z., Han X., Liu X., Yang L., Huang C., Miao Y. (2021). Potentiation of Plant Defense by Bacterial Outer Membrane Vesicles Is Mediated by Membrane Nanodomains. Plant Cell.

[38] Rutter B.D., Innes R.W. (2017). Extracellular Vesicles Isolated from the Leaf Apoplast Carry Stress-Response Proteins. Plant Physiol..

[39] Rutter B.D., Innes R.W. (2018). Extracellular Vesicles as Key Mediators of Plant–Microbe Interactions. Curr. Opin. Plant Biol..

[40] Raffaele S., Bayer E., Lafarge D., Cluzet S., German Retana S., Boubekeur T., Leborgne-Castel N., Carde J.-P., Lherminier J., Noirot E. (2009). Remorin, a Solanaceae Protein Resident in Membrane Rafts and Plasmodesmata, Impairs *Potato Virus X* Movement. Plant Cell.

[41] Son S., Oh C.J., An C.S. (2014). Arabidopsis Thaliana Remorins Interact with SnRK1 and Play a Role in Susceptibility to Beet Curly Top Virus and Beet Severe Curly Top Virus. Plant Pathol. J..

[42] Fu S., Xu Y., Li C., Li Y., Wu J., Zhou X. (2018). Rice Stripe Virus Interferes with S-Acylation of Remorin and Induces Its Autophagic Degradation to Facilitate Virus Infection. Mol. Plant.

[43] Brault M.L., Petit J.D., Immel F., Nicolas W.J., Glavier M., Brocard L., Gaston A., Fouché M., Hawkins T.J., Crowet J. (2019). Multiple C2 Domains and Transmembrane Region Proteins (MCTP s) Tether Membranes at Plasmodesmata. EMBO Rep..

[44] Beauchemin C., Bougie V., Laliberte J.F. (2005). Simultaneous Production of Two Foreign Proteins from a Polyvirus-Based Vector. Virus Res..

[45] Ivanov K.I., Puustinen P., Gabrenaite R., Vihinen H., Ronnstrand L., Valmu L., Kalkkinen N., Makinen K. (2003). Phosphorylation of the Potyvirus Capsid Protein by Protein Kinase CK2 and Its Relevance for Virus Infection. Plant Cell.

[46] German-Retana S., Candresse T., Alias E., Delbos R., Le Gall O. (2000). Effects of GFP or GUS Tagging on the Accumulation and Pathogenicity of a Resistance Breaking LMV Isolate in Susceptible and Resistant Lettuce Cultivars. Mol. Plant-Microbe Interact..

[47] Ito Y., Esnay N., Platre M.P., Wattelet-Boyer V., Noack L.C., Fougère L., Menzel W., Claverol S., Fouillen L., Moreau P. (2021). Sphingolipids Mediate Polar Sorting of PIN2 through Phosphoinositide Consumption at the Trans-Golgi Network. Nat. Commun..

[48] Simon P. (2003). Q-Gene: Processing Quantitative Real-Time RT-PCR Data. Bioinformatics.

[49] Bernard A., Domergue F., Pascal S., Jetter R., Renne C., Faure J.-D., Haslam R.P., Napier J.A., Lessire R., Joubes J. (2012). Reconstitution of Plant Alkane Biosynthesis in Yeast Demonstrates That Arabidopsis ECERIFERUM1 and ECERIFERUM3 Are Core Components of a Very-Long-Chain Alkane Synthesis Complex. Plant Cell.

[50] Martin K., Kopperud K., Chakrabarty R., Banerjee R., Brooks R., Goodin M.M. (2009). Transient Expression in *Nicotiana benthamiana* Fluorescent Marker Lines Provides Enhanced Definition of Protein Localization, Movement and Interactions *in Planta*. Plant J..

[51] Legrand A., Martinez D., Grélard A., Berbon M., Morvan E., Tawani A., Loquet A., Mongrand S., Habenstein B. (2019). Nanodomain Clustering of the Plant Protein Remorin by Solid-State NMR. Front. Mol. Biosci..

[52] Martinez D., Legrand A., Gronnier J., Decossas M., Gouguet P., Lambert O., Berbon M., Verron L., Grélard A., Germain V. (2019). Coiled-Coil Oligomerization Controls Localization of the Plasma Membrane REMORINs. J. Struct. Biol..

[53] Roberts I.M., Wang D., Findlay K., Maule A.J. (1998). Ultrastructural and Temporal Observations of the Potyvirus Cylindrical Inclusions (Cls) Show That the Cl Protein Acts Transiently in Aiding Virus Movement. Virology.

[54] Sasaki N., Takashima E., Nyunoya H. (2018). Altered Subcellular Localization of a Tobacco Membrane Raft-Associated Remorin Protein by Tobamovirus Infection and Transient Expression of Viral Replication and Movement Proteins. Front. Plant Sci..

[55] Perraki A., Binaghi M., Mecchia M.A., Gronnier J., German-Retana S., Mongrand S., Bayer E., Zelada A.M., Germain V. (2014). StRemorin1.3 Hampers *Potato Virus X* TGBp1 Ability to Increase Plasmodesmata Permeability, but Does Not Interfere with Its Silencing Suppressor Activity. FEBS Lett..

[56] De Storme N., Geelen D. (2014). Callose Homeostasis at Plasmodesmata: Molecular Regulators and Developmental Relevance. Front. Plant Sci..

[57] Zavaliev R., Levy A., Gera A., Epel B.L. (2013). Subcellular Dynamics and Role of *Arabidopsis* β-1,3-Glucanases in Cell-to-Cell Movement of Tobamoviruses. Mol. Plant-Microbe Interact..

[58] Wan J., Laliberté J.-F. (2015). Membrane-Associated Virus Replication Complexes Locate to Plant Conducting Tubes. Plant Signal. Behav..

