# Supplementary material for: StREM1.3 REMORIN Protein Plays an Agonistic Role in Potyvirus Cell-to-Cell Movement in N. benthamiana
Source: Viruses. 2022 Mar 10;14(3):574. doi: 10.3390/v14030574 (PMC8951588; doi:10.3390/v14030574)
Supplement: Supplementary file 1 [file viruses-14-00574-s001.zip › viruses-1611309-Supplementary Figures.pdf]

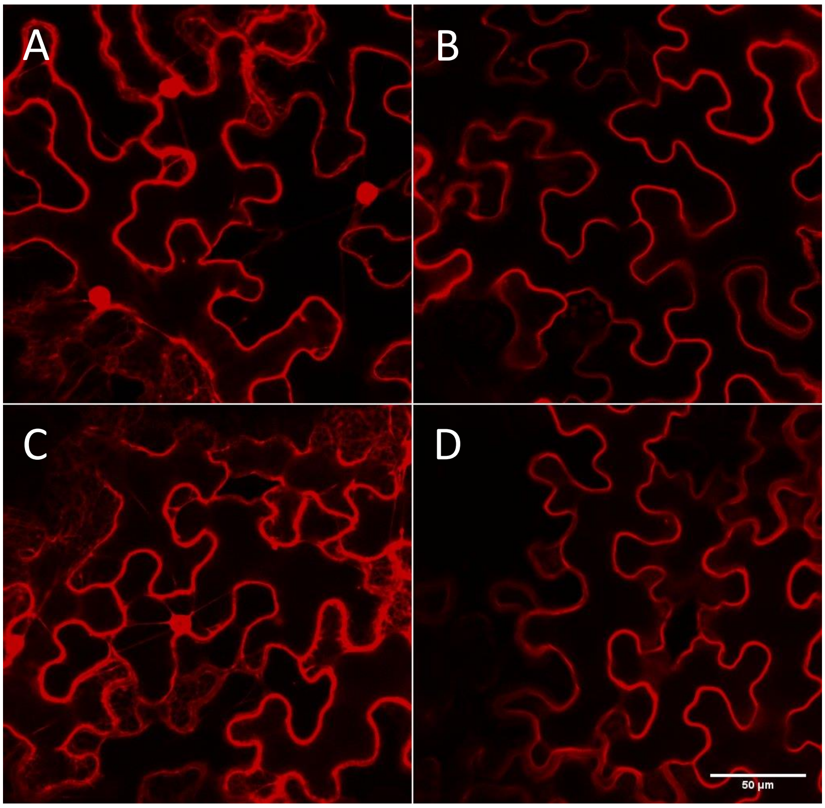

<u>**Supplemental Figure S1.**</u> **Confocal microscopy images representative of the subcellular localization of transiently expressed proteins 3 days after agroinfiltration**. (**A**) RFP (**B**) RFP-StREM1.3 (**C**) RFP-StREM1.3$^{1-170}$ (**D**) RFP-StREM1.3^{-AAA}. *N. benthamiana* epidermis cell expressing RFP and RFP-StREM1.3$^{1-170}$ show labeling distribution through the whole cell (cytosol and nucleus) whereas RFP-StREM1.3 and RFP-StREM1.3^{-AAA} display labeling at the PM only. Scale bar: 50 µm.

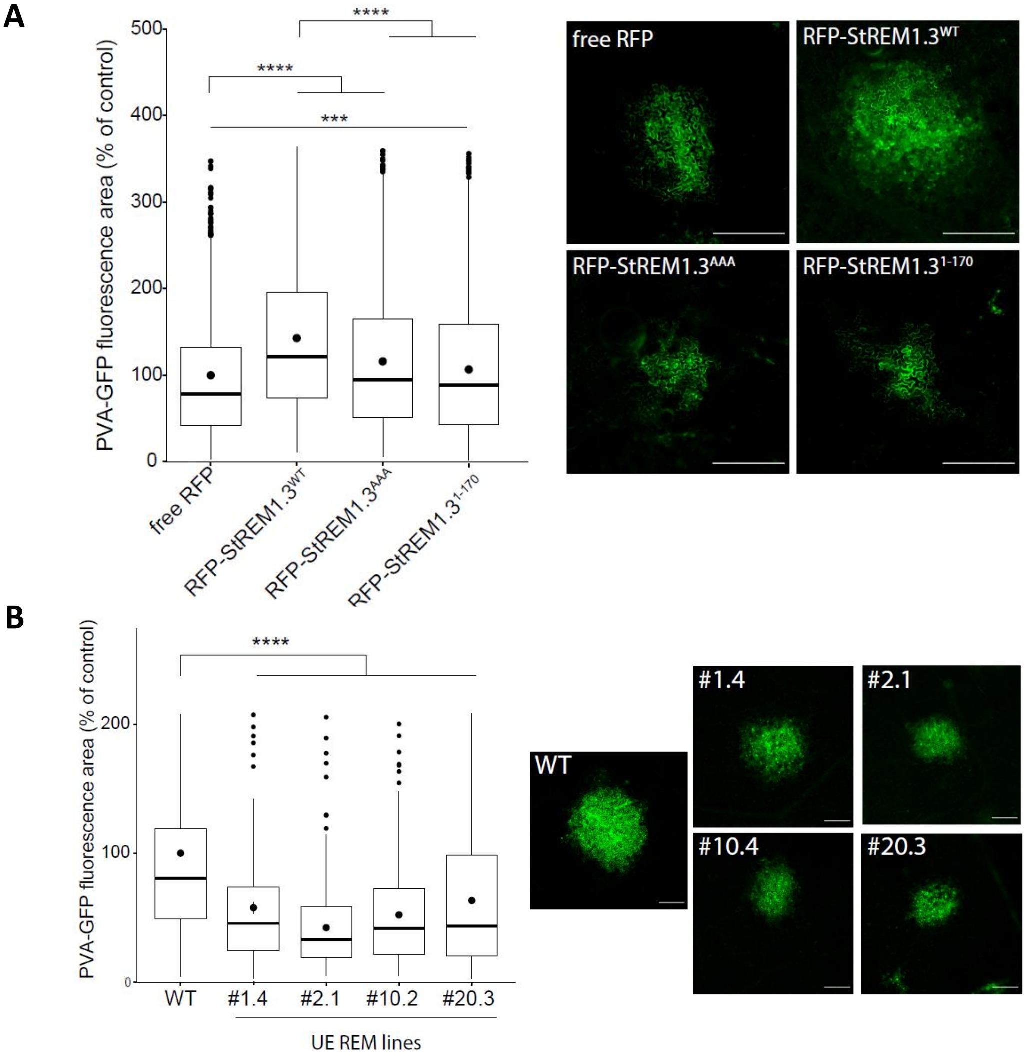

<u>Supplemental Figure S2.</u> (**A**) **Effect of transient over-expression of stREM1.3 and its mutant forms on PVA spreading in *N. benthamiana*.** The area of GFP infection foci is normalized and expressed in % of the area of PVA propagation in the absence of REM (free RFP negative control). At least 1000 GFP infection foci from at least 5 independent experiments were imaged at 3 days post-inoculation (dpi). Statistical analysis was performed using one-way ANOVA followed by Student's t-test in R software (Anova, $p < 2.2e{-16}$:**** <0.001, , ***:0.001). (**B**) **PVA spreading is slowed down in four independent transgenic *N. benthamiana* lines constitutively expressing hairpin REM constructs (Under expressing lines, UE REM lines).** The area of infection foci is normalized and expressed in % of the area of PVA propagation in WT plants. At least 100 infection foci from at least 4 independent repeats were imaged at 3 days post-inoculation (dpi). Statistical analysis was performed using one-way ANOVA followed by Student's t-test in R software (Anova, $p < 2.2e{-16}$:**** <0.001). Scale bar: 500 µm.

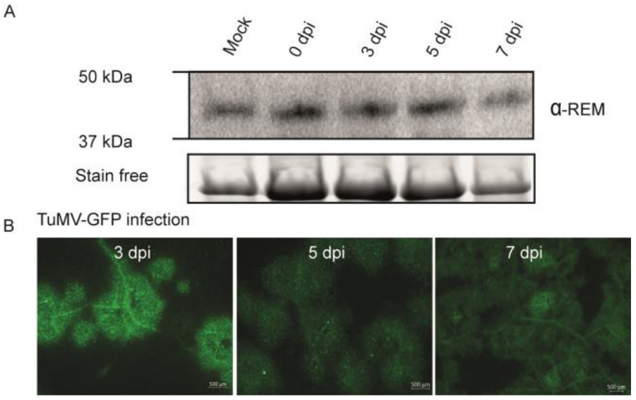

<u>Supplemental Figure S3.</u> **The levels of endogenous *N. benthamiana* REMs are not modified by TuMV-GFP infection.** (**A**) Western blot against endogenous NbREM was performed on total protein extracts from inoculated *N. benthamiana* leaves infected by TuMV-GFP at 0, 3, 5 and 7 dpi. Stain free loading is indicated below (**B**) Representative images of the corresponding inoculated leaves. Bar=500 µm

---

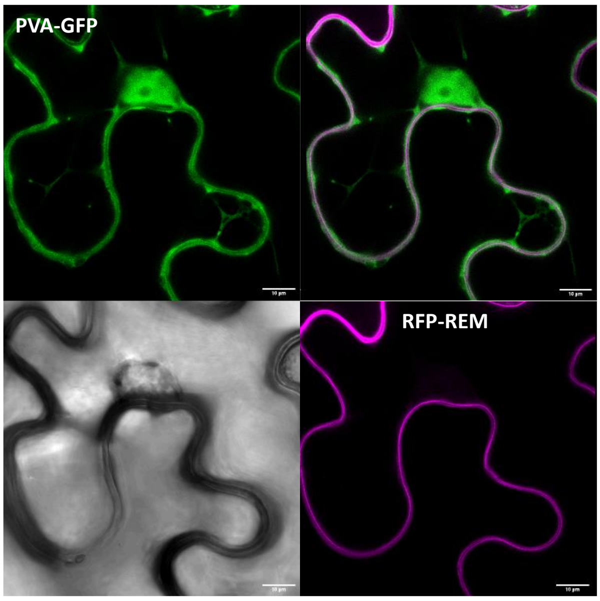

<u>Supplemental Figure S4.</u> **PVA infection does not modify StREM1.3 membranous localization.** Representative confocal mages showing the membranous transiently expressed RFP-StREM1.3 in *N. benthamiana* leaf epidermal cells infected by PVA-GFP at 5 dpi. The pink signal labels the RFP-StREM1.3 that remains membranous. The green signal labels the cytoplasm and nucleus and corresponds to free GFP expressed during PVA-GFP infection. At least two independent experiments were performed. Scale bar: 10 µm.

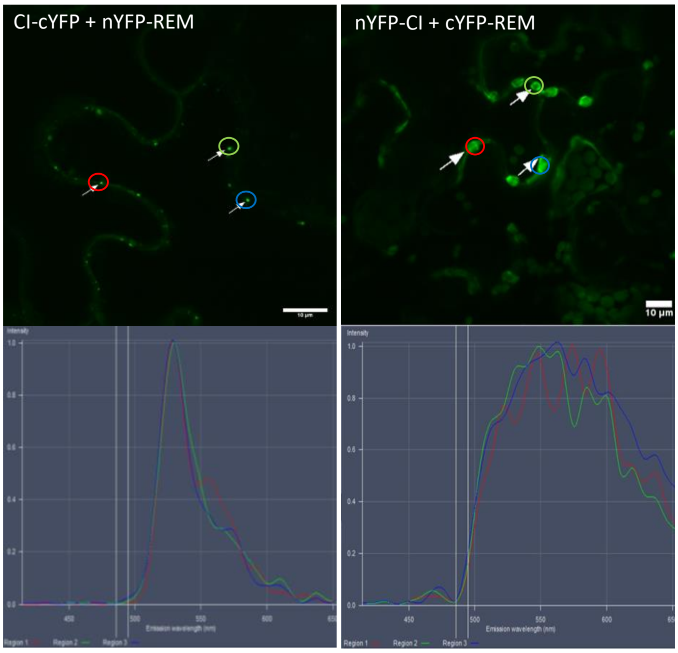

<u>Supplemental Figure S5</u>: Use of the Lambda scan mode to confirm the nature of fluorescence emission signal in BiFC experiments. Left: positive signal (nYFP-REM and CI-cYFP). Three ROI spots (region of interest) are indicated by the arrows. The emission spectrum of colored ROIs within 400-650nm wavelength range is indicated for each ROI. A maximum of fluorescence emission is observed at 530 nm which corresponds to YFP emission wavelength. Right: negative signal (cYFP-REM and nYFP-CI). No fluorescence emission is observed for each of the ROIs at 530 nm.

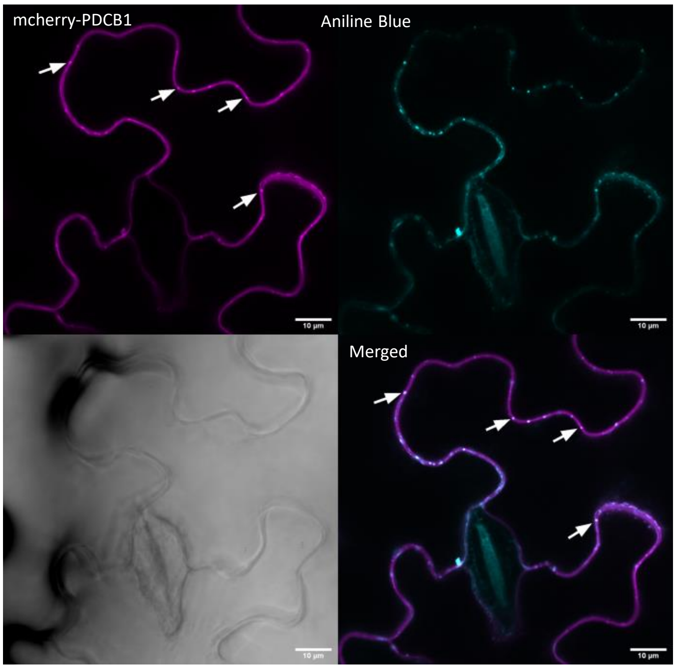

<u>Supplemental Figure S6</u>: Representative confocal image showing aniline blue staining of callose deposition at the PD pitfields in *N. benthamiana* leaf cells expressing mcherry-PDCB1. Callose deposition is labelled in blue. White arrows indicate co-labeled spots in the PM. Scale bar: 10 µm.
